# On the learning benefits of confidence-weighted testing

**DOI:** 10.1186/s41235-016-0003-x

**Published:** 2016-09-22

**Authors:** Erin M. Sparck, Elizabeth Ligon Bjork, Robert A. Bjork

**Affiliations:** Department of Psychology, University of California, Los Angeles, 1285 Franz Hall, Box 951563, Los Angeles, CA 90095 USA

**Keywords:** Memory, Learning, Testing effects, Multiple-choice, Confidence ratings

## Abstract

Taking multiple-choice practice tests with competitive incorrect alternatives can enhance performance on related but different questions appearing on a later cued-recall test (Little et al., Psychol Sci 23:1337–1344, 2012). This benefit of multiple-choice testing, which does not occur when the practice test is a cued-recall test, appears attributable to participants attempting to retrieve not only why the correct alternative is correct but also why the other alternatives are incorrect. The present research was designed to examine whether a confidence-weighted multiple-choice format in which test-takers were allowed to indicate their relative confidence in the correctness of one alternative compared with the others (Bruno, J Econ Educ 20:5–22, 1989; Bruno, Item banking: Interactive testing and self-assessment: Volume 112 of NATO ASI Series, pp. 190–209, 1993) might increase the extent to which participants engaged in such productive retrievals. In two experiments, such confidence-weighted practice tests led to greater benefits in the ability of test-takers to answer new but related questions than did standard multiple-choice practice tests. These results point to ways to make multiple-choice testing a more powerful tool for learning.

## Significance

How taking tests affects the retention of information that is not itself directly tested, but is related to the directly tested information, is an important question for the field of education, in part because questions on practice quizzes are rarely repeated verbatim on later final examinations. Well-constructed multiple-choice practice tests can improve the recall of such related information on a final examination by encouraging test-takers to activate information in memory about incorrect alternatives in order to reject them. Not all test-takers engage in such a productive strategy, but the present findings suggest that a new confidence-weighted multiple-choice testing format may encourage more test-takers to do so, thus indicating how to construct more effective learning materials.

## Background

The act of retrieving information is a powerful learning event. The retrieval process itself changes recalled information, making it more easily accessible in the future (e.g., Bjork, 1975; Carrier & Pashler, 1992). Actively retrieving information on a test, even without feedback, results in better long-term retention of that material than does restudying that information, a phenomenon known as the *testing effect* (for reviews, see Dempster, 1996; Roediger & Karpicke, 2006). Test-taking can thus serve as a valuable pedagogical tool—not just as a tool for assessment.

Multiple-choice tests, though perhaps the most ubiquitous type of test, are often criticized as being less effective as tools for learning than are more open-ended test formats, such as short-answer, cued-recall, and free-recall tests. Such criticism arises primarily because multiple-choice tests are thought to rely more on recognition processes and/or fail to induce the kinds of retrieval processes that can support later retention (Carpenter & DeLosh, 2006; Foos & Fisher, 1988; Glover, 1989; Hamaker, 1986). Recent research, however, has demonstrated that well-constructed multiple-choice tests can trigger productive retrieval processes (Little, 2011; Little & Bjork, 2010, 2015; Little, Bjork, Bjork, & Angello, 2012), not only about why the correct answer is correct but also about why an incorrect alternative is incorrect. As a consequence, performance on a later question to which a previously incorrect alternative is now the correct answer can be enhanced, even when the later test is a cued-recall test. Importantly, this same benefit is not found if the initial question is a cued-recall question (Little et al., 2012).

The triggering of such productive retrieval processes with respect to incorrect alternatives as well as correct alternatives is contingent on the incorrect alternatives being competitive (Little & Bjork, 2015). *Uranus*, for example, would be a competitive incorrect alternative to the question, “Which outer planet was discovered by mathematics rather than direct observation?” because Uranus is an outer planet. In contrast, *Mercury* would be a noncompetitive incorrect alternative for this question because it can be rejected easily, given that it is an inner planet. Importantly, however, although the presence of competitive alternatives appears to be a necessary condition for eliciting retrieval as to why a given incorrect alternative is incorrect, it does not appear to be a sufficient condition: Little (2011) found that only about 30 % of test-takers engage in such a strategy without being explicitly instructed to do so.

Our goal in the present research was to see if test-takers could be led to engage to a greater extent in such productive retrieval processes with respect to incorrect alternatives without their being explicitly instructed to do so. More specifically, we wondered if a format in which test-takers were encouraged to select answers by deliberatively assessing their confidence in a given answer relative to the other alternatives might trigger more productive retrieval processes than does a standard multiple-choice format. To test this idea, we used a confidence-weighted form of multiple-choice testing developed by Bruno (1989, 1993).

**Fig. 1:**
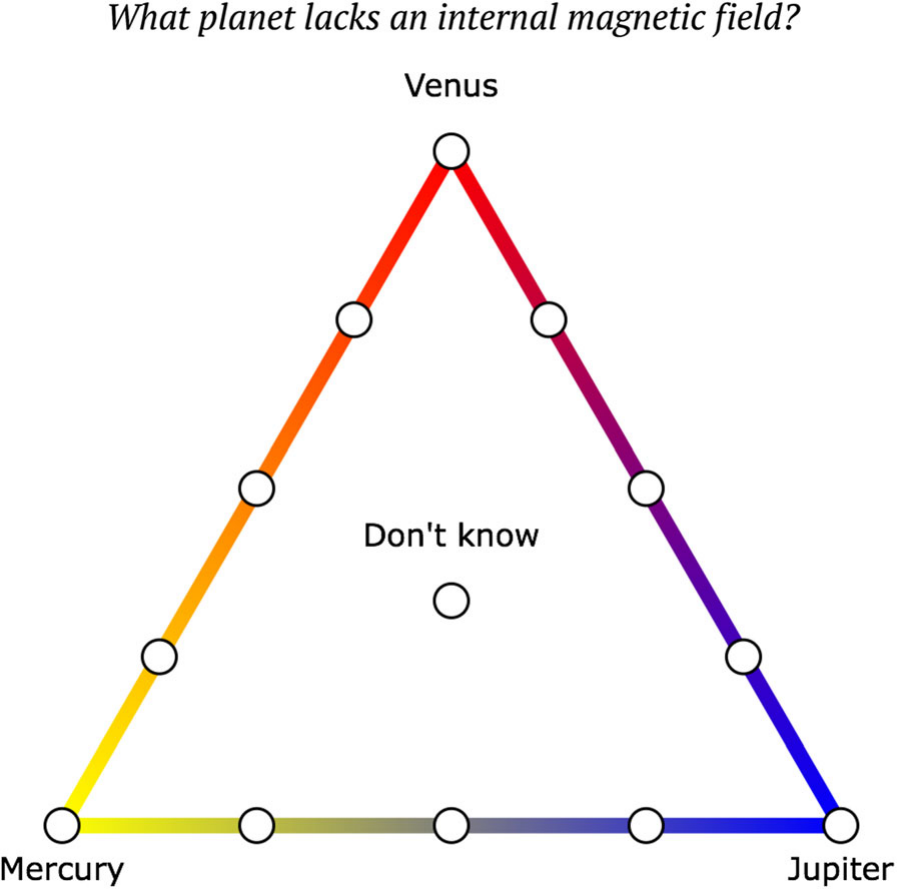
Example of a confidence-weighted multiple-choice item with the alternative answer choices appearing on the vertices

In this format, three alternatives are placed at the corners or vertices of a triangle (e.g., *Venus*, *Mercury*, and *Saturn*) as the possible answers to the question, “What planet lacks an internal magnetic field?” as illustrated in Fig. 1. Test-takers can select one of these alternatives (say, *Venus*), or, instead, they can select an intermediary point along one of the lines connecting two vertices of the triangle (e.g., along the line connecting the answers *Venus* and *Mercury*). Selection of one of the alternatives at a vertex of the triangle indicates complete confidence in that answer. Selection at one of the intermediary points along the line connecting two vertices of the triangle indicates uncertainty with respect to which of those alternatives is the correct answer and certainty that the alternative on the other side of the triangle is incorrect. In addition, the format allows a test-taker to indicate his or her relative confidence in the correctness of each of the alternatives that seem plausible. To illustrate, if the test-taker believes either *Venus* or *Mercury* could be correct but is more confident that *Venus* is correct, he or she can select a point along the line connecting *Venus* and *Mercury* that is closer to *Venus* than to *Mercury*. In short, this format allows for partial knowledge to be demonstrated: Selecting a point between *Venus* and *Mercury* indicates that the test-taker has confidently rejected *Saturn* as the correct answer, and selecting a point along the line between *Venus* and *Mercury* that is closer to *Venus* than *Mercury* indicates that the test-taker believes *Venus* is more likely to be the correct answer than is *Mercury*.

**Fig. 2:**
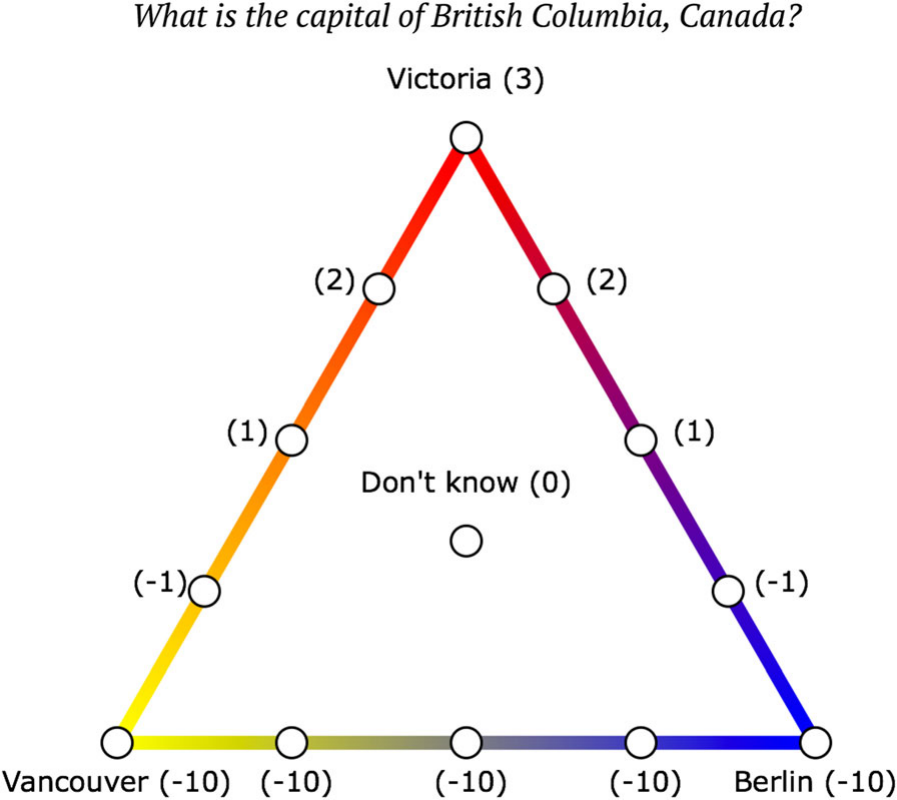
Example of a confidence-weighted multiple-choice question used to instruct participants in the use of this format. Test-takers can select any of the circles as their answer, and the number of points that would be gained or lost for each answer is shown in parentheses next to the corresponding circle, given that *Victoria* is the correct answer to this question. Highly confident incorrect answers at the vertices and along the line between the two incorrect answers are highly penalized, while incorrect answers along the sides of the triangle connected to the correct answer are only marginally penalized, relative to the points received for answering the question correctly with high confidence

Scoring with this format differs from that of a standard multiple-choice test in important ways. First, in terms of how points are assigned, guessing is greatly discouraged: Choosing an incorrect alternative, or choosing any point on the line between the two incorrect alternatives, which amounts to rejecting the correct answer, results in a major loss of points (10 points in our research and as shown in Fig. 2, which illustrates the question we used for instructing participants in the use of the confidence-weighted multiple-choice format). In addition, a test-taker has the option of choosing the point in the middle of the triangle, which means that the question is worth 0 points (i.e., the test-taker will neither lose nor gain points by choosing this option). Finally, as shown for the example question illustrated in Fig. 2, the number of points gained for choosing a correct alternative is only marginally greater than that for choosing points which are near that alternative on either of the sides which include that alternative.

## Experiment 1

In Experiment 1, we compared performance on a final cued-recall test of studied passages that were followed by (a) an initial confidence-weighted multiple-choice test, (b) a standard multiple-choice test, or (c) no initial test (i.e., a study-only condition). Given that our primary goal was to see if an initial multiple-choice test using the confidence-weighted format—versus the standard multiple-choice format—would lead to more retrieval of why an incorrect alternative was not the correct answer, we restricted the questions on the final cued-recall test to questions for which the correct answer had appeared earlier as an incorrect alternative in the initial tests given to participants in the two testing conditions.

### Methods

#### Participants

A total of 150 undergraduates (107 women, *M*_age_ = 20.3 years) at the University of California, Los Angeles, recruited online from the Sona subject pool participated for partial course credit. All participants were fluent in English. An a priori power analysis was conducted using G*Power software to determine a sufficient sample size using a medium effect size (*f* = 0.25), an alpha of 0.05, and a power of 0.80. On the basis of this analysis, we aimed for a total sample size of 159. Our actual sample, however, fell a few participants short, owing to the end of the academic year.

#### Design and materials

Participants were randomly assigned to one of the two testing conditions (i.e., standard multiple-choice or confidence-weighted multiple choice) or the no-test condition (i.e., the study-only condition). In all three conditions, participants studied two passages, one on the planet Saturn and one on Yellowstone National Park, which were passages taken from the materials used by Little et al. (2012). Each passage was about 1100 words in length, and the order in which they were studied was counterbalanced across participants.

Those participants randomly assigned to one of the two test conditions took either a computer-paced 10-item standard multiple-choice test (lasting 4.17 min, 25 s per question) following their reading of each passage or a 10-item confidence-weighted multiple-choice test (lasting 4.17 min, 25 s per question) following their reading of each passage. So, across the two passages, participants answered a total of 20 questions. To ensure that sufficient time was given to process information about all of the alternatives, participants in both testing conditions were not allowed to advance the questions on their own, but instead had to spend the 25-s period with each question, as had been done in the previous research of Little et al. (2012). Participants assigned to the no-test condition played the Tetris computer game for the same length of time as it took to complete the multiple-choice tests given in the other two conditions (i.e., 4.17 min) to ensure that the final cued-recall test occurred with the same delay duration for all participants. Performance of the participants randomly assigned to the no-test (i.e., study-only) condition served as the baseline against which to compare the performance of participants in the other two conditions.

The type of initial test experienced was manipulated as a between-subjects variable to control for the possibility of carryover effects that might influence a participant’s strategy; that is, if a participant took a test using the confidence-weighted format following the first passage, he or she might be more likely to engage in a different, perhaps more thoughtful strategy when tested on the second passage with a standard multiple-choice test.

Questions appearing on the initial and final tests were constructed from ten pairs of related multiple-choice test questions of equivalent difficulty, taken from the materials used by Little et al. (2012), by randomly selecting one question from each pair to be on the initial test and the other to be on the later cued-recall test. An example of one such pair (based on the passage about Yellowstone National Park) is shown in Table 1. As illustrated there, the two questions in a pair (denoted A and B) were always about the same topic (e.g., about geysers in Yellowstone National Park in this particular example), and both questions were presented with the same alternative choices (e.g., *Steamboat Geyser*, *Old Faithful*, and *Castle Geyser*). For question A, however, the correct answer is *Steamboat Geyser*, and for Question (B), the correct answer is *Castle Geyser*. To construct the two tests, the A or B question in each pair was randomly selected to be on the initial multiple-choice test or to appear as the related but not previously tested question on the final cued-recall test. When a question appeared on the final cued-recall test, no alternatives were presented along with the question. On each test, the questions appeared in random order, and which test was given as the initial test or as the final cued-recall test was counterbalanced across participants.

**Table 1:** Example Question Pair with Corresponding Correct and Incorrect Alternatives

Example question pair	Alternatives	
	Correct	Incorrect
(A) What is the tallest geyser in Yellowstone National Park?	Steamboat Geyser	Castle Geyser Old Faithful
(B) What is thought to be the oldest geyser in Yellowstone National Park?	Castle Geyser	Steamboat Geyser Old Faithful

### Procedure

Before reading any passages, participants randomly assigned to be in the confidence-weighted test condition were instructed how to answer questions on the triangle-shaped scale using confidence to guide their answer selection, and they were told how the scoring of performance with this type of format differed from that of a standard multiple-choice test. How to mark the answer they considered best on the basis of their confidence was demonstrated using the question, “What is the capital of British Columbia, Canada?” as illustrated in Fig. 2. Participants were told that test-takers using this type of multiple-choice format would proceed as follows. If completely confident in the answer *Victoria*, the test-taker would mark the circle at the vertex of the triangle where Victoria appears. Because the correct answer to this example question is, in fact, *Victoria*, this response would receive 3 points. If the test-taker indicated complete confidence in the answer being *Vancouver*, however, and thus marked the circle at the vertex of the triangle where *Vancouver* appears, which is incorrect, the test-taker would be penalized 10 points. Similarly, selecting any answer lying on the line between the two incorrect alternatives (i.e., *Vancouver* and *Berlin* in this example) would also lose 10 points. If the test-taker was quite confident that the correct answer was Victoria but thought there was at least some possibility it might be *Vancouver*, then the test-taker should mark the circle along the line between *Victoria* and *Vancouver* that is closest to the *Victoria* vertex of the triangle. Marking that specific circle would gain 2 points. If the test-taker was evenly split in one’s confidence between *Victoria* and *Vancouver* being the correct answer, then the test-taker should mark the circle halfway between the *Victoria* and *Vancouver* vertices. Given that one of these choices is correct (i.e., *Victoria* in this example), marking that circle would receive 1 point. Marking the circle on this line closest to *Vancouver*, however, would lose 1 point. If the test-taker was completely unsure about the correct answer and thought that any of them could be correct, then the test-taker could mark the *Don’t know* circle in the middle of the triangle and no points would be awarded nor lost, but the instructions and nature of the scoring system discouraged participants from selecting this option. Participants were told that the coloration of the lines in the triangle was just another way to illustrate the relationships between answer choices (i.e., halfway between red and yellow is orange), but that the coloration was nondiagnostic of the correct answer.

Following the presentation of these instructions, participants had to complete a tutorial and correctly answer a series of questions to check that they understood the directions regarding how to use this different test format and how it would be scored before they were allowed to continue. Participants in the standard multiple-choice test condition were simply instructed to read and answer questions when they appeared on the screen as they normally would when taking a multiple-choice test.

Similarly to the procedure used by Little et al. (2012), participants next read a passage for 9 min and then answered questions or played the Tetris computer game, depending on their experimental condition. Participants repeated these steps for the second passage, reading then answering questions or playing Tetris. After answering all of the questions on each initial test, participants in the testing conditions were told their summary score for that initial test, mainly to encourage participants in the confidence-weighted testing condition to choose intermediary points in the face of uncertainty and to avoid making highly confident errors. Participants were not given any specific item-by-item feedback as to which questions were answered correctly or incorrectly.

After completion of the last initial multiple-choice test, participants in the two test conditions then played Tetris for 5 min while the participants in the study-only condition continued to play Tetris for another 5 min. Then, all participants received a 20-item (10 items per passage) final cued-recall test. For participants in the study-only condition, all questions appearing on the final cued-recall test were new. For participants in the two test conditions, all questions on the final cued-recall test were also new, but each one was related to a question they had previously answered on the initial multiple-choice tests. That is, unlike in the previous study by Little et al. (2012), no questions on the final cued-recall test of the present Experiment 1 were identical to ones asked on the initial multiple-choice tests. Specifically, questions for each participant came from the other member of the question pair (e.g., Question B in Table 1 if Question A had appeared on the initial test for that participant). All questions on the final test, however, were displayed without alternatives, as the final test was a cued-recall test. Given the construction of the question pairs from which the initial and final tests were built, as previously described, the correct answer to each question on the final cued-recall test had always appeared as a competitive incorrect alternative to a question previously answered on one of the initial multiple-choice tests for participants in either of the test conditions.

On the final cued-recall test, passages were tested in the same order as they had been read. Questions were blocked by passage, but their order was randomized within that block. The final cued-recall test, unlike the initial tests, was self-paced. Participants were encouraged to attempt to answer all questions even if they were unsure of their answers. No penalties were assessed for incorrect answers on the final test. Answers on the final cued-recall test were scored according to a guide that allowed leniency for spelling mistakes.

At the conclusion of Experiment 1, all participants were asked open-ended survey questions regarding strategies used during the administered tests and whether they had any comments about the study. Additionally, participants in the confidence-weighted multiple-choice condition were shown a series of statements (see Appendix for a complete list) regarding their opinions of the initial confidence-weighted tests. These were scored on a 5-point Likert scale to assess metacognitive awareness, with response choices ranging from 1 (Strongly disagree) to 5 (Strongly agree). All participants were debriefed and thanked for their time and participation.

### Results and Discussion

#### Initial-test performance

Initial test scores for participants in the standard multiple-choice condition and the confidence-weighted multiple-choice condition are not directly comparable, as they are calculated in different ways. We do report and compare initial test performance for the first and second tests, respectively, within each testing condition as a way of gauging whether participants’ test-taking strategies changed between their taking of the first test and their taking of the second test. We thought it possible, for example, that participants in the confidence-weighted multiple-choice condition—after seeing their scores on the first initial test—might switch to a more conservative strategy between tests by, say, using the intermediary points more frequently on the second initial test. Such a change in strategy might then, in turn, lead to performance differences for the related questions asked about each passage on the final test, perhaps resulting in our seeing a benefit for related items only from the second passage.

For participants in the standard multiple-choice test condition, performance obtained on the first passage (*M* = 6.48, *SD* = 1.88) did not differ significantly from that obtained on the second passage [*M* = 6.98, *SD* = 1.86; *t*(49) = 1.58, *p* = .12]. Similarly, for participants in the confidence-weighted multiple-choice condition, the average score obtained on the test of the first passage (*M* = 4.82, *SD* = 17.11) did not differ significantly from that obtained on the test of the second passage [*M* = 4.16, *SD* = 18.29; *t*(49) = .22, *p* = .83]. Thus, in neither of the initial test conditions was there an indication that participants changed their test-taking strategies from the first to the second test. Hence, in our analyses of final-test performance, we have collapsed participants’ performance across the two passages. With respect to the issue of whether participants did take advantage of the opportunity allowed by the confidence-weighted testing format to indicate partial knowledge about a question, individuals in this condition did demonstrate partial knowledge 19.8 % of the time (i.e., receiving 2, 1, or −1 points on some questions).

#### Final-test performance

**Fig. 3:**
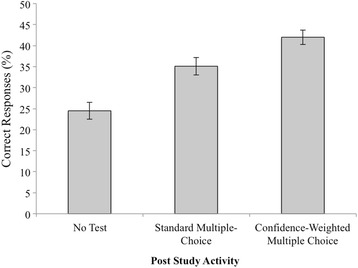
Correct performance on related questions on the final cued-recall test for each poststudy activity (no test, standard multiple-choice test, and confidence-weighted multiple-choice test) in Experiment 1. Error bars represent ±1 SEM

Averages for correct performance on the final cued-recall test, calculated on the basis of 20 items (10 from each passage), were 4.92 (*SD* = 2.81) items (24.5 %) for participants in the study-only group; 7.02 items (*SD* = 2.59) (35.1 %) for those in the standard-multiple choice group; and 8.4 (*SD* = 2.5) items (42 %) and for those in the confidence-weighted multiple-choice condition (Fig. 3). As indicated in Fig. 3, and as confirmed by a one-way analysis of variance (ANOVA), we observed a significant overall effect of initial activity following the reading of each passage (Tetris, standard multiple-choice test, or confidence-weighted multiple-choice test) on final test performance [*F*(2,147) = 20.59, *p* < .001, *η* ^*2*^ = .22]. Planned comparison *t* tests between the standard multiple-choice condition and the study-only baseline condition [*t*(98) = 3.68, *p* < .001, *d* = .74] and between the confidence-weighted multiple-choice condition and the study-only baseline condition [*t*(98) = 6.631, *p* < .001, *d* = 1.33] indicate a significant testing effect for both types of multiple-choice tests relative to no test, findings that are in line with the previous literature. Importantly, however, given the primary question of the present research, a planned comparison *t* test between the two multiple-choice conditions revealed that participants taking confidence-weighted multiple-choice tests following their reading of the passages were then able to answer related questions on the final cued-recall test significantly better than did the participants who took a standard multiple-choice test [*t*(98) = 2.54, *p* = .01, *d* = .51].

#### Survey results (confidence-weighted multiple-choice group)

Participants in the confidence-weighted multiple-choice test condition indicated that they understood the instructions on how to use this different test format (*M* = 4.26, median = 4, mode = 4), that they generally felt the confidence-weighted format assessed their knowledge more effectively relative to a standard multiple-choice test (*M* = 3.7; median = 4; mode = 4), and that they liked being able to receive partial credit using the confidence-weighted multiple-choice format (*M* = 4.25, median = 4, mode = 4).

Most participants stated that the initial test made them think more carefully about their answers on the final test than they would have without having been exposed to the initial test (*M* = 3.88, median = 4, mode = 4). Additionally, as we hoped might be the case, most participants also stated that they recalled information from the passage about the incorrect alternatives when selecting their answers on the initial test (*M* = 3.49, median = 4, mode = 4). However, despite stating that they did recall information about the incorrect alternatives, most participants did not indicate that taking the initial test had necessarily helped them perform well on the final test (*M* = 2.92, median = 3, mode = 2), as compared with not having taken an initial test at all, even though final test performance indicates otherwise.

That most participants reported recalling information about incorrect alternatives while taking the confidence-weighted multiple-choice test, but also did not report feeling that taking the initial test had allowed them to perform better on the final test than they would have done otherwise, indicates what might be called a lack of *metacognitive sophistication* on their part regarding their learning and is in line with much recent evidence suggesting that students do not tend to appreciate which types of study activity best foster learning, despite their many years of active involvement in both formal and informal learning activities (for a discussion of this view and the relevant research, see, e.g., Bjork & Bjork, 2015; Bjork, Dunlosky, & Kornell, 2013). To be fair to the present participants, however, it needs to be noted that the present experiment did not give them much experience with this new type of multiple-choice format and also did not afford them a direct comparison of their performance on the final test following both types of prior multiple-choice testing formats. It is possible that more experience with using this new type of multiple-choice format, plus more specific feedback regarding the enhanced later test performance that it fosters, might eventually lead test-takers to become more metacognitively aware of how it is serving to enhance their later test performance.

## Experiment 2

Although the results observed on the final cued-recall test in Experiment 1 were consistent with our expectations that a confidence-weighted multiple-choice testing format would induce test-takers to engage in more retrieval of information associated with incorrect alternatives, which would in turn lead to a greater ability to answer related questions on the final cued-recall test, it is possible that this difference is not attributable to the use of the confidence-weighted multiple-choice testing format per se. Whereas we believe it is the relational confidence judgments among alternatives that test-takers are encouraged to make with this format that underlie their enhanced performance on the later cued-recall test, it is possible that the effect is driven by participants’ simply making a confidence judgment with respect to their choice on a given question. We tested this possibility in Experiment 2 by comparing the final cued-recall test performance of participants who, following their reading of text passages, took initial multiple-choice tests using (a) the standard format, (b) the standard format plus confidence judgments, or (c) the confidence-weighted format.

### Methods

#### Participants

A total of 114 undergraduates (87 women, *M*_age_ = 20.2 years) at the University of California, Los Angeles, recruited online from the Sona subject pool, participated for partial course credit. One participant’s data in the confidence-weighted multiple-choice condition and one participant’s data in the standard multiple-choice condition were excluded from analysis because these individuals did not follow instructions. The data from one participant in the standard multiple-choice plus confidence-judgment condition was also excluded from analysis, owing to an error in recording. All participants were fluent in English. An a priori power was conducted using G*Power software to determine a sufficient sample size with a medium effect size (*f* = 0.3), an alpha of 0.05, and a power of 0.80. On the basis of this analysis, we had aimed for a total sample size of 111.

#### Design and materials

The design of Experiment 2 was the same as that for Experiment 1, except for the introduction of a standard multiple-choice plus confidence-judgment condition and the deletion of a study-only group. The materials for all groups were the same as those used in Experiment 1.

### Procedure

For the confidence-weighted multiple-choice and standard multiple-choice conditions, the procedure remained the same as in Experiment 1. The procedure for the standard multiple-choice plus confidence-judgment condition added the query, “On a scale of 0–100 (where 0 is not at all and 100 is completely), how confident are you in your answer?” in a text box appearing after each multiple-choice question. Participants typed in their response to this query after answering each initial standard multiple-choice test question. The initial multiple-choice test question and all of the alternatives stayed on the screen along with a filled-in mark next to the answer selected by the participant. All of the text was grayed out, except for the confidence-judgment question. Participants were not allowed to change their selected answer once the initial 25-s presentation duration had expired and the confidence-judgment query appeared on the computer screen. Once these participants had typed a number into the confidence-judgment text box, a button appeared on the screen allowing them to move on to the next question. The Tetris distractor task and the final cued-recall test consisting of related questions remained the same.

### Results and Discussion

#### Initial-test performance

As we did for Experiment 1, we report initial test performance on the first versus the second initial test within each testing condition, not for making comparisons between the two testing conditions, but as a way to assess whether participants’ test-taking strategies, particularly in the confidence-weighted multiple-choice condition, changed between the first initial test and the second initial test. In the standard multiple-choice condition, performance on the test of the first passage (*M* = 7, *SD* = 2.05) did not differ significantly from that on the second passage [*M* = 7.43, *SD* = 1.83; *t*(36) = −1.37, *p* = .18]. Similarly, in the standard multiple-choice plus confidence-judgment condition, performance on the test of the first passage (*M* = 6.57, *SD* = 1.94) did not differ significantly from that on the test of the second passage [*M* = 7.14, *SD* = 1.87; *t*(36) = −1.5, *p* = .14]. Finally, in the confidence-weighted multiple-choice condition, average scores on the test of the first passage (*M* = −6.05, *SD* = 20.23) did not significantly differ from those on the test of the second passage (*M* = 1.08, *SD* = 16.95; *t*(36) = −1.82, *p* = .08), suggesting, as in Experiment 1, that participants did not change their test-taking strategies from their first to their second tests. Because these comparisons did not indicate such a change, we again collapsed scores across the two passages into our analyses of final test performance.

With respect to the issue of whether individuals in the confidence-weighted testing condition took advantage of the opportunity this format allowed for demonstrating partial knowledge about a question, these participants—similar to their Experiment 1 counterparts—demonstrated partial knowledge 20.8 % of the time (i.e., receiving 2, 1, or −1 points for some of the questions).

Finally, although initial test performance in the confidence-weighted multiple-choice condition in Experiment 2 is numerically lower than that observed in Experiment 1 (*M* = 4.82 and 4.16), we believe this difference is due to the large variability in the scores, which is greatly affected by marking even a single highly confident but incorrect answer. Additionally, Experiment 2 scores are within 1 SD of Experiment 1 scores.

#### Final-test performance

**Fig. 4:**
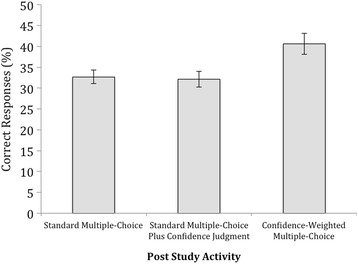
Correct performance obtained for related answers on the final cued-recall test as a function of initial test type (standard multiple-choice, standard multiple choice plus confidence-judgment, confidence-weighted multiple choice) in Experiment 2. Error bars represent ±1 SEM

Average correct performance on the final cued-recall test, based on a total of 20 items (10 items from each passage), for participants in the standard multiple-choice, standard multiple-choice plus confidence judgment, and confidence-weighted multiple-choice conditions, respectively, was 6.54 (*SD* = 2.14) items (32.7 %), 6.41 (*SD* = 2.85) items (32.1 %), and 8.11 (*SD* = 2.34) items (40.6 %) (Fig. 4). As indicated in Fig. 4 and confirmed by one-way ANOVA, we obtained a significant overall effect of initial activity following reading of each passage (standard multiple-choice, standard multiple-choice plus confidence-judgment, or confidence-weighted multiple-choice test) on final test performance [*F*(2,108) = 5.46, *p* = .006, *η* ^*2*^ = .09].

Replicating the findings of Experiment 1, we found the taking of initial confidence-weighted multiple-choice tests led to better final cued-recall test performance for related information than did the taking of initial standard multiple-choice tests [*t*(72) = −3, *p* = .004]. New to Experiment 2, and consistent with our belief that it is the making of relational confidence judgments among alternatives invoked by the confidence-weighted multiple-choice testing format that gives rise to participants’ enhanced performance on the later cued-recall test rather than just the making of a confidence judgment about the correctness of answer choices per se, we also found that (a) the taking of initial confidence-weighted tests produced superior final cued-recall performance than did the taking of initial standard multiple-choice tests with confidence judgments added [*t*(72) = 2.97, *p* = .004], and that (b) final cued-recall performance obtained in the standard multiple-choice condition and performance obtained in the standard multiple-choice plus confidence condition did not differ significantly [*t*(72) = .45, *p* = .65]. Thus, it would seem that making a numeric confidence judgment in the correctness of one’s final answer choice without relating it to the other alternatives does not provide a retention boost to the nontested, related information, leading to performance similar to that on a standard multiple-choice test.

## General Discussion

As shown by Little et al. (2012), multiple-choice tests can be effective in activating information that is not directly tested, making retrieval of this information easier in the future. More specifically, those researchers showed that answering standard-format multiple-choice questions containing competitive incorrect alternatives (i.e., plausible answers) enhanced participants’ ability to answer questions based on such related but nontested information on a later cued-recall test. The pattern of results obtained in the present research supports that conclusion and additionally demonstrates that asking multiple-choice questions in a confidence-weighted format can increase such benefits.

### Limitations

As previously mentioned, we believe it is the making of relational confidence judgments among alternatives that test-takers are encouraged to do in the confidence-weighted multiple-choice format that underlies their enhanced performance on the later cued-recall test as compared with participants taking either a standard initial multiple-choice tests or no initial tests at all. Experiment 2 served to further support this explanation of why initial confidence-weighted multiple-choice tests lead to higher performance for related questions than do initial standard multiple-choice tests by showing that the additional requirement of having to make a global confidence judgment regarding one’s answer choice in the standard multiple-choice test format does not provide the same enhancement for the retention of related information.

Other differences between the confidence-weighted multiple-choice format and the standard multiple-choice format exist as well, however, and could possibly also contribute, at least to some degree, to the greater benefit for retention of untested related information observed with this testing format in the present experiments. For example, the inclusion of a *Don’t know* option in the confidence-weighted format, used in both the present research and in Bruno’s (1989, 1993) original format, might play a role in the production of this benefit. Although this option was selected only 8.7 % of the time across both of the present experiments, and thus its availability seems unlikely to have been a major factor affecting the observed pattern of results, it is nonetheless possible that just its presence as a response option could have impacted the type of processing in which participants engaged when taking such multiple-choice tests. We speculate, however, that, rather than increasing participants’ engagement in the type of productive retrieval processes we believe to underlie the benefit for retention of related information observed by Little et al. (2012) and in the present research, its presence may have been counteractive. Perhaps, for example, the presence of a *Don’t know* option acts something like a bailout option for when test-takers immediately think they do not know the answer. They can just quickly select it rather than being forced to think about their confidence in each alternative relative to the others. In short, the presence of a *Don’t know* option might occasionally rob test-takers of the opportunity to strengthen what they know via a critical evaluation of all the information they can retrieve regarding each of the alternatives.

Another difference between the standard multiple-choice and confidence-weighted multiple choice testing formats is the point values associated with incorrect answers. The assessing of a large penalty for a highly confident incorrect answer in the confidence-weighted multiple-choice format (i.e., losing 10 points from your score) may well encourage different types of processing from those occurring in the standard multiple-choice format, where typically—rather than being penalized for selecting a wrong answer—one just does not receive any points for a wrong answer. Individual differences in risk-taking and risk aversion, for example, could contribute to the adoption of slightly different test-taking strategies in the two types of formats. The point values used in the confidence-weighted testing format employed in the present research were selected to maintain its similarities to Bruno’s (1989, 1993) original format, in which points were assigned in a way that should strongly *discourage* guessing so that errors could be viewed by instructors as reflecting confusion or misunderstanding, not guessing, with respect to the topic of the question. To this end, point values are assigned so as to encourage students—in the face of uncertainty—to use intermediary points along the lines connecting the vertices of the triangle. More specifically, the scoring procedure used in both the present experiments and those of Bruno results in the differences in points *earned* being minimal for choosing the correct answer at its vertex versus choosing an intermediary point along one of the lines connecting it to the other vertices; in contrast, the differences in points *lost* for choosing an incorrect answer at its vertex versus choosing an intermediary point along those same lines is great. How the use of different point values, or even the exclusion of points all together, might affect the processing strategies in which test-takers engage in the confidence-weighted testing format could well be a promising line for future research.

Although we employed one way to control for the variable of time on task in the present research—namely, by equating the presentation time for all questions across the different types of initial tests for all participants (similar to the procedure of Little et al., 2012, where participants were given 24 s to answer each standard multiple-choice question), one could well argue that this type of procedure for controlling time on task does not necessarily control for *functional* time on task across the different types of testing formats explored in the present research. If, for example, participants begin to mind-wander after the selection of their answers, and the average time to arrive at a selection tends to be less in the standard multiple-choice format than in the confidence-weighted multiple-choice format, it could well be that participants are spending less time engaged in productive processing when taking the former versus the latter type of multiple-choice test. Allowing participants to proceed in a self-paced manner while taking the different types of multiple-choice tests would shed light on this theoretical question. From a practical standpoint, however, the present results already indicate that, in situations in which students are allotted the same amount of time to take practice multiple-choice tests, giving students confidence-weighted multiple-choice tests rather than standard multiple-choice tests is a more effective use of that time.

Finally, although the present research is focused on the positive consequences that the taking of initial or practice multiple-choice tests can have on later test-taking performance, it is important to note that there can also be potential negative effects of taking initial or practice multiple-choice tests on later test-taking performance, such as the persistence of endorsed misinformation or errors from the initial tests, particularly when corrective feedback is not provided or is delayed (e.g., Butler, Marsh, Goode, & Roediger, 2006; Roediger & Marsh, 2005). The number of alternatives presented during initial tests and their difficulty can also affect later retention of the tested information (e.g., Whitten & Leonard, 1980). Careful construction of the initial test is thus an important consideration when employing a multiple-choice test of any format as a potential learning tool.

## Conclusions

Overall, our findings suggest that confidence-weighted multiple-choice tests can be particularly useful as practice tests, especially to the extent that questions on the subsequent test (the one that matters or matters more) are different questions. It also seems possible that consistent use of the confidence-weighted format might lead to students’ becoming, in general, more appreciative of or sensitive to the benefits of retrieval practice. Survey research by Kornell and Bjork (2007) and Hartwig and Dunlosky (2012) on students’ study strategies has demonstrated that students are aware of one benefit of testing—namely, that testing themselves can identify better than can restudying what they have and have not learned and understood—but that they are largely unaware that the act of retrieval itself can be a learning event in the sense that the retrieved information becomes more retrievable in the future than it would have been otherwise. Although in the present Experiment 1 participants, when questioned, seemed largely unaware that answering questions using the confidence-weighted multiple-choice format strengthened their knowledge of information related to both correct and incorrect alternatives, perhaps more extensive practice with this testing format would result in learners’ becoming more metacognitively sophisticated about the benefits of trying actively to retrieve what has been studied.

If so, then experience gained in using the confidence-weighted multiple-choice format might well change the degree to which students engage in productive retrieval about all alternatives when taking standard multiple-choice tests as well, allowing them to maximize the benefits of either type of test-taking practice for their performance on later examinations. Should such generalization occur, instructors would not always be constrained to using the confidence-weighted multiple-choice format, which does take more time to create and also to score, to optimize their students’ learning. After training their students in the use of the confidence-weighted format, instructors could then switch back to using the more traditional multiple-choice format.

## Appendix

1. I understood the formatting.
2. I liked being able to receive partial credit.
3. The format better assessed what I knew compared with a standard multiple-choice test.
4. The initial test made me think more carefully about my answers on the final test than I otherwise would have.
5. I recalled information from the passage about the incorrect alternatives when selecting my answer on the initial test.
6. Taking the initial test helped me answer more questions on the final test than if I had not used it.
7. Taking the initial test made me more confident in my answers on the final test.

These items are scored as follows: 1 = Strongly disagree, 2 = Disagree, 3 = Neutral, 4 = Agree, and 5 = Strongly agree.
